# Lessons from history for $\dot{V}O_{2}$ max and the $\dot{V}O_{2}$ plateau, part 1, 1920 – 1961: original concepts were based on discontinuous exercise protocols

**DOI:** 10.3389/fphys.2025.1688750

**Published:** 2025-12-05

**Authors:** Robert A. Robergs, Bridgette O’Malley, Sam Torrens, Craig Ryan McNulty, Praneel Titheradge, Julien S. Baker, Todd A. Astorino, Simon Green, Marek Nalos

**Affiliations:** 1 School of Exercise and Nutrition Sciences, Faculty of Health, Queensland University of Technology, Brisbane, QLD, Australia; 2 Faculty of Health Studies, Jan Evangelista Purkyne University, Usti nad Labem, Czechia; 3 Department Of Sports Medicine and Active Health Sciences, Faculty of Medicine, Charles University, Plzen, Czechia; 4 School of Allied Health, Exercise and Sports Sciences, Charles Sturt University – Port Macquarie, Port Macquarie, NSW, Australia; 5 Fellow of the Asia Pacific Technological Sciences Academy (FAPTSA), Hong Kong Baptist University, Hong Kong, Hong Kong SAR, China; 6 Department of Kinesiology, California State University-San Marcos, San Marcos, CA, United States; 7 Sport and Exercise Science, School Of Health Sciences, Western Sydney University-Campbelltown Campus, Campbelltown, NSW, Australia; 8 ICU, 1st Department of Internal Medicine I, Plzen University Hospital and Charles University, Plzen, Czechia

**Keywords:** oxygen consumption, cycling, running, incremental exercise, research methodology, dogma

## Abstract

**Purpose:**

The maximal rate of oxygen uptake ( $\dot{V}O_{2}$ max) has an early history (1920–1961) based on discontinuous incremental exercise protocols. Regardless, debate continues on many sub-topics and methodologies involved in this measure. There could be lessons to learn about the relevance, or not, of content within the accumulating knowledge of this topic if there is a detailed account of the research of this time-period.

**Methods:**

Manuscript references were retrieved from PubMed and Google Scholar for the targeted topics and time-period.

**Results:**

In 1923 and 1924, Hill proposed that during discontinuous incremental exercise bouts, there is eventually a levelling in $\dot{V}O_{2}$ despite increasing exercise intensity or sustained effort. Subsequent researchers in the 1950’s described this ‘levelling in $\dot{V}O_{2}$ ’ observation as a plateau, which functioned to verify $\dot{V}O_{2}$ max. However, when critiquing the data from studies with valid methodology, evidence of a $\dot{V}O_{2}$ plateau at or near $\dot{V}O_{2}$ max was only seen in a subset of participants in 1924 (2 of 7), with added evidence in 1959 (2 of 4) and 1961 (4 of 5). Collectively, 50% of the subjects were unable to attain a $\dot{V}O_{2}$ plateau response at $\dot{V}O_{2}$ max.

**Conclusion:**

Despite major limitations to the published research and data interpretations prior to 1961, such work led to the incorrect (not evidence-based) expectation that all participants should demonstrate a $\dot{V}O_{2}$ plateau at or near $\dot{V}O_{2}$ max. The inter-connectedness of $\dot{V}O_{2}$ max and the $\dot{V}O_{2}$ plateau concepts thereby became engrained into the pre-1970s, and perhaps later, epistemology of exercise physiology.

## Introduction

We are now past the 100-year anniversary of the publication of Hill and Lupton’s manuscript on the supply and utilization of oxygen (O_2_) during exercise (Hill and Lupton, 1923), and the 1924, 8-part further elaboration of this work (Hill et al., 1924a; Hill et al., 1924b; Hill et al., 1924c). Consequently, it is timely to critically reflect on the last 100 years of research evidence on each of the exercise physiology of O_2_ consumption ( $\dot{V}O_{2}$ ) with increments in exercise intensity, maximal $\dot{V}O_{2}$ ( $\dot{V}O_{2}$ max) and the presence, or not, of a plateau in $\dot{V}O_{2}$ at or near $\dot{V}O_{2}$ max.

It is important to acknowledge the pioneering research that set the historical precedent of any discipline, in addition to the researchers who had the inquisitiveness at that time to research the then unknown responses of the human body to exercise stress. Many researchers of this early history of exercise physiology had to build their own exercise equipment and measurement instrumentation, and compute by hand their statistical analyses. Based on these issues, it is remarkable what such research was able to accomplish. Yet, as with all research of any discipline, there are limitations, and occasionally mistakes. The awareness of the limitations and identification of any mistakes are important as they provide the opportunity for the refinement of methods and/or data interpretations, which are both essential for progress to be made.

One hundred years is a long period of time and is connected to considerable prior research. Within this time-period, numerous scientists have written review manuscripts that have focussed on specific aspects of this history in their more contemporary interpretations of the exercise physiology of $\dot{V}O_{2}$ , $\dot{V}O_{2}$ max and the presence, or not, of a plateau in $\dot{V}O_{2}$ at or near $\dot{V}O_{2}$ max (Howley et al., 1995; Noakes, 1997; Bassett and Howley, 2000; Bassett, 2002; Noakes, 2008; Niemeyer et al., 2021; Burtscher et al., 2023; Millet et al., 2023). Regardless, such work, as well as others focussed on mechanisms of the determinants to $\dot{V}O_{2}$ max (Brink-Elfegoun et al., 2007; Hawkins et al., 2007; Levine, 2008; Ferretti, 2014; Lundby et al., 2017; Hoppeler, 2018; Joyner and Dominelli, 2021; Wagner, 2023) have limited relevance to this review simply because they were not concerned with a detailed critical assessment of all research linked directly, or indirectly, to the initial research methodology of $\dot{V}O_{2}$ max and the $\dot{V}O_{2}$ plateau at $\dot{V}O_{2}$ max.

Why is this early research so important? Because the impact of Hill’s introduction of the concept of both $\dot{V}O_{2}$ max and the $\dot{V}O_{2}$ plateau at $\dot{V}O_{2}$ max as definitive proof that there is a $\dot{V}O_{2}$ that cannot be exceeded despite an increase in exercise intensity, is still influential within contemporary exercise physiology research and professional practice in current time. The best example of this is how Noakes used the expectation of how all individuals should demonstrate a $\dot{V}O_{2}$ plateau at $\dot{V}O_{2}$ max, but where research evidence reveals otherwise, to propose that there must be other determinants to the need to terminate exercise than cardio-pulmonary limitations linked to the constrained ability to continue to increase $\dot{V}O_{2}$ (Noakes, 1997; Noakes, 2011). Such logic framed the initial explanation of the Central Governor Model (Robergs, 2017).

But what if a detailed account of the early history proves that the concept of the expectation that all individuals should demonstrate a $\dot{V}O_{2}$ plateau at $\dot{V}O_{2}$ max was wrong? What if the research conducted in the transition from the use of discontinuous incremental exercise protocols to continuous incremental exercise protocols was not adequate in establishing the construct validity of the $\dot{V}O_{2}$ plateau at $\dot{V}O_{2}$ max applied to continuous incremental exercise testing? How would such errors have influenced the research that followed? What problems have arisen in the interpretation of this research because of these potential flaws? What has been the influence of these interpretations across the multiple decades of research that followed?

### The purpose and explanations of structure

When persistent conjecture surrounds a topic, especially when it spans more than 100 years, it is logical to re-assess the historical record. Such a reassessment is necessary to discern whether, in this passage of time, features of the original research have been overlooked, interpretations have been incorrect, or important aspects within the subsequent pursuit of science have not been adhered to. The presence of such deficiencies could influence some of the discrepancies and divergent opinions on numerous other topics linked to $\dot{V}O_{2}$ max and the $\dot{V}O_{2}$ plateau at $\dot{V}O_{2}$ max.

Critical inquiry, and especially that involving historical reflection, is a vitally important part of science. The writings of both Kuhn (1962) and Popper (1995) document the scope of potential error that can be incorporated into the pursuit of science, and where a large proportion of this error might occur unintentionally by scientists themselves. For example, as stated by Popper (1995), “*I do admit that at any moment we are prisoners caught in the framework of our theories; our expectations; our past experiences; our language.*” [p. 56.] Such a quote translates into our past education, for it is this education that has imposed the theories, expectations and learning experiences that culminates in the foundation for how we view and understand our discipline knowledge. The unavoidable reality is that this learning occurs in conjunction with that of our teachers, which then, of course, is influenced by added prior decades of inquiry, adding further risk for the possible entrenchment of these multiple layers of misinterpretation within the conventional understanding. Such practice, when devoid of critical thinking and conjecture, yields the uncomfortable label of dogma.

Consequently, the intent of this endeavour is to provide an in-depth critical inquiry of the historical development of the concepts of $\dot{V}O_{2}$ max and the $\dot{V}O_{2}$ plateau. This is a large undertaking, for as previously explained, such work must span more than 100 years of prior research inquiry. This cannot be done in one manuscript. Consequently, this manuscript is Part-1 of a 4-part series that explores, in detail, the key aspects of prior research on these topics from 1920 to 1961, which ends at the time of the transition from discontinuous to continuous incremental exercise protocols. Future parts of this series will follow research inquiry into the use and influence of improved instrumentation, and the transition to continuous incremental exercise protocols (e.g., step and ramp incremental exercise) and the added research inquiry connected to them (Part-2: 1960–1990). Added content will cover the progression to more contemporary topics and the related gained knowledge that are pertinent to progressive improvement in the measurement and interpretation of $\dot{V}O_{2}$ max and the $\dot{V}O_{2}$ plateau (Part-3: 1990 to present). Part-4 will then provide a more fully informed (based on the new knowledge gained from this historical reflection) overview of the findings and the related evidence they reveal for further research of specific topics, and the need for possible revised thinking and data interpretations of $\dot{V}O_{2}$ max and the $\dot{V}O_{2}$ plateau, and the varied topics linked to these measures. It is important for the reader to understand that such historical research inquiry is firmly based on the core concepts of the methodologies and data interpretations of $\dot{V}O_{2}$ max and the $\dot{V}O_{2}$ plateau. Similarly, the content of this Part-1, and other Parts, will be confined to the period of time of the manuscript. For this manuscript, this means that to have a fresh assessment of the research there needs to be a focus on just the research of this period. Yes, we are informed by the wealth of research since this time, and that can aid in the re-assessment of the prior work. However, the time to reveal and cite more recent research occurs in Part-2 and -3. We believe that this approach allows us to identify the strengths and weaknesses of the research of the time period, as well as transfer this gained knowledge to future research to ascertain the benefit or misinformation that the results of this work might reveal. We will apply this approach across all Parts of this series.

## An overview of the research: 1920–1961

The period of history from 1920 – 1961 is important for exercise physiology and related disciplines in that it witnessed the beginnings of the concepts of $\dot{V}O_{2}$ max and the $\dot{V}O_{2}$ plateau, occurred prior to the digital revolution, and where research of $\dot{V}O_{2}$ max and the $\dot{V}O_{2}$ plateau were based on the use of discontinuous, incremental exercise protocols. Such research was explored using the PubMed™ database for the stated time period using criterion words or terms of $\dot{V}O_{2}$ max, maximal oxygen consumption, aerobic capacity, exercise, treadmill running, and cycling. Further refinement of the literature searches occurred using Google Scholar and the reference lists of the accumulating research manuscripts. This research described how to measure and understand $\dot{V}O_{2}$ max in the context of this time period, and considerable methods and results derived from discontinuous exercise protocols of this period can be scrutinized to ascertain the limitations and/or strengths of such procedures and their physiological understanding.

Discontinuous exercise protocols are those that involve single bouts of exercise to steady state or exhaustion, depending on the intensity. Their use for measuring $\dot{V}O_{2}$ max involves the completion of added exercise bouts, after sufficient recovery, but of a higher exercise intensity. This process is continued and eventually data obtained for the peak $\dot{V}O_{2}$ for each exercise bout is graphed to document a profile for peak $\dot{V}O_{2}$ vs. exercise intensity. This review will also use historical findings pertinent to $\dot{V}O_{2}$ max and the $\dot{V}O_{2}$ plateau to develop theories and/or hypotheses (none of which were developed in research manuscripts of these early years) to highlight progressions in research and establish what issues have or have not been suitably addressed.

This historical review will show that scientists from 1920 to 1961 used what would now be referred to as inappropriate methodology, misinterpreted data, presented data incorrectly, failed to challenge proposed assumptions, avoided the crafting of theories, and overlooked essential data from other research publications that could have altered the directions, in important ways, of the subsequent research on these topics throughout, and beyond, this time-period.

The review will end with a comprehensive summary of the knowledge gained from this period, identification of the pertinent good and bad features of this work, and what this thorough review of the research of this time period means to research, practice and knowledge of $\dot{V}O_{2}$ max and the $\dot{V}O_{2}$ plateau in current time.

### The multi-generational impact of AV Hill’s data interpretations on VO_2_max and the VO_2_ plateau

To begin with, comment will be directed once again at the impact of the research of A.V. Hill within exercise physiology, and in particular, the concepts of $\dot{V}O_{2}$ max and the $\dot{V}O_{2}$ plateau. Such work has been the focus of prior reviews, though not all have included detailed content on the developmental history of $\dot{V}O_{2}$ max and the $\dot{V}O_{2}$ plateau. The most pertinent recent historical accounts of the early research on $\dot{V}O_{2}$ max and the $\dot{V}O_{2}$ plateau, especially by A.V. Hill, has been that of Noakes (1997), Noakes (2008), Noakes (2011), Bassett and Howley (2000), Bassett (2002) and Niemeyer et al. (2021). Specific details of this scholarship, as they relate to $\dot{V}O_{2}$ max and the $\dot{V}O_{2}$ plateau, follows.

It is important to acknowledge that Noakes (1997) initially presented evidence in one of the five ‘ugly and creaking edifices’ of $\dot{V}O_{2}$ max, for how Hill did not present evidence, due to deficient methodology, for demonstrating the presence of a $\dot{V}O_{2}$ plateau at $\dot{V}O_{2}$ max. Noakes summarized such an interpretation of Hill’s prior research and supposed documentation of a $\dot{V}O_{2}$ plateau response, with the comment, “*their major conclusion that VO*_*2*_ *reaches a plateau during exercise of progressively increasing intensity was not proven because this test of refutation was not conducted*.” In this context, the deficient test of refutation was the lack of a $\dot{V}O_{2}$ measure during the final minute of an additional exercise about, at a higher intensity than the prior bout of exercise completed by Hill. However, Noakes overlooked the more detailed data presented by Hill et al. (1924c) where data was reported for 7 subjects, and where only two of these subjects revealed a levelling in the $\dot{V}O_{2}$ response across discontinuous exercise bouts of increasing exercise intensity. As such, Hill et al. (1924c) did present evidence of a subset of subjects (2 of 7 subjects) who may have attained a $\dot{V}O_{2}$ plateau at $\dot{V}O_{2}$ max, but which was far from the evidence needed to support the blanket assumption that all subjects should attain this response for the $\dot{V}O_{2}$ max value to be valid. In contrast, the main question, based on the evidence, that required raising and answering was why all subjects did not demonstrate a $\dot{V}O_{2}$ plateau at $\dot{V}O_{2}$ max!

Consequently, it could be stated that Hill and Lupton (1923) and Hill et al. (1924c) introduced the concept of a $\dot{V}O_{2}$ plateau at $\dot{V}O_{2}$ max, where future research was therefore tasked with the need to clarify, define, verify and/or refute the concept. Noakes commented on only one other research study of that early era in his critical commentary of this edifice, which was that of Mitchell et al. (1958). However, as this review will document, there were multiple other studies that required emphasis and explanation due to their being of far greater importance and impact to the developing historical or traditional narrative of the $\dot{V}O_{2}$ plateau at $\dot{V}O_{2}$ max.

Noakes (2008) further added to his historic accounts of Hill’s research by now recognizing the data of Hill et al. (1924c), providing further conceptual arguments surrounding the research intent and purpose of Hill and Lupton (1923) and Hill et al. (1924c), and more pertinently, to respond to a prior commentary by Howley (2007). A brief coverage of the research of Taylor et al. (1955) was provided, but as this review will show, the Taylor et al. study required a far more detailed summary due to the impact it has had on decades of research that followed on $\dot{V}O_{2}$ max and the $\dot{V}O_{2}$ plateau, and especially the methodological definition of the $\dot{V}O_{2}$ plateau. Such detail will be provided in this review.

Bassett and Howley (Bassett and Howley, 2000) included a section on the historical development of $\dot{V}O_{2}$ max in their review of the factors that limit $\dot{V}O_{2}$ max. This section (Part I, page 70–72) presented a relatively concise summary of Hill’s research, with added support from a selection of more contemporary research that further contributed to the topic of the review, most notably that of Astrand and Saltin (1961b) and Duncan et al. (1997). As previously explained, this content is constrained by the limited coverage of the early research of $\dot{V}O_{2}$ max that occurred between 1920 and 1961, as will be shown by the content of this review.

Bassett (2002) provided a summary of the collective life and research of A.V Hill. This is a remarkably valuable collection of the expanse of the research contributions of A.V. Hill to numerous topics of physiology. The content of the review included attention to the concepts of $\dot{V}O_{2}$ max and the $\dot{V}O_{2}$ plateau (see pages 1572–1575), but such work was constrained in scientific relevance to the topic of this review because of the broader scope of their purpose. More work and critical historical evidence-based research inquiry is needed to complete such coverage. We provide that in this review. Finally, Niemeyer et al. (2021) completed a thorough review of the factors that might influence the development of a $\dot{V}O_{2}$ plateau at $\dot{V}O_{2}$ max. Yet as with Bassett (2002), the historical content was brief, the early research of the topic was not critically challenged, and there was an underlying bias in the acceptance of the expectation that all subjects should demonstrate a $\dot{V}O_{2}$ plateau at $\dot{V}O_{2}$ max. There were added features of this review that were open to constructive refinement, and these issues will be addressed in Part-3.

The added rationale for this historical investigation of the research from 1920 to 1961, which represents Part-1 of a 4-part series, is largely revealed in an understanding of more contemporary research, interpretation, and commentary. The details of this later work will be presented in Parts-2 and -3. Yet for now it is important to note that in the last 50 years, the topic of the temporal response of $\dot{V}O_{2}$ during continuous incremental exercise has continued to be widely investigated, with a clear emphasis on the coincident attainment of $\dot{V}O_{2}$ max, the $\dot{V}O_{2}$ plateau at $\dot{V}O_{2}$ max, and the dilemmas that occur (or not) when such coincident development does not occur (Bassett, 2002; Bassett and Howley, 2000; Burtscher et al., 2023; Day et al., 2003; Duncan et al., 1997; Howley et al., 1995; Howley, 2007; Millet et al., 2023; Noakes, 1997; Noakes, 2008; Poole and Jones, 2017).

Despite the prior research and commentary on $\dot{V}O_{2}$ max and the $\dot{V}O_{2}$ plateau, which is dependent on the physiological determinants to the temporal response of $\dot{V}O_{2}$ during incremental exercise that precedes $\dot{V}O_{2}$ max, only limited critical research inquiry has occurred on the $\dot{V}O_{2}$ to time (or intensity) profile and the between subject differences of this response. The traditional or historic narrative on this issue is represented by acceptance of three main observations and related understanding; 1. skeletal muscle dominates the $\dot{V}O_{2}$ response to exercise; 2. cardiopulmonary (O_2_ delivery) and skeletal muscle endurance (O_2_ extraction/consumption) adaptations dominate the determinants for muscle $\dot{V}O_{2}$ ; and 3. there is a linear relationship between whole body (from pulmonary gas exchange) $\dot{V}O_{2}$ and exercise intensity during incremental exercise to exhaustion. While some commentary (Poole and Jones, 2017) and research (Aaron et al., 1992a; b; Iannetta et al., 2019; Keir et al., 2016; Kipp et al., 2024; Korzeniewsky, 2018; Marks et al., 2005; O’Malley et al., 2024; Vella et al., 2006; Zoldaz et al., 1995) have provided evidence that could be interpreted as anomalies to the historical narrative, such evidence has yet to change methodologies used in the mainstream measurement and interpretation of $\dot{V}O_{2}$ max and the $\dot{V}O_{2}$ plateau.

The irony of this oversight is that you cannot interpret a change in the slope of the temporal change in $\dot{V}O_{2}$ ( $\dot{V}O_{2}$ gain) near $\dot{V}O_{2}$ max (the $\dot{V}O_{2}$ plateau) if there is no research evidence-based understanding of the changing $\dot{V}O_{2}$ gain (increasing or decreasing) that precedes $\dot{V}O_{2}$ max and the underlying physiological responses that contribute to these changes. Consequently, it is no surprise to observe that not only is there deficient understanding of the changing $\dot{V}O_{2}$ gain during incremental exercise, but also continued conjecture, and for some topics eventual clarity, involving decades of added research on the exercise protocols to use (and not use) to measure $\dot{V}O_{2}$ max (Astrand and Saltin, 1961b; Astorino et al., 2004; Beltz et al., 2016; Buchfuhrer et al., 1983; Yoon et al., 2007), how to define $\dot{V}O_{2}$ max (Hawkins et al., 2007; Poole and Jones, 2017; Robergs, 2017), how to process data to minimize variability and quantify $\dot{V}O_{2}$ max (Martin-Ricon et al., 2019; Nolte et al., 2023; Robergs et al., 2010), what the definition of a $\dot{V}O_{2}$ plateau at $\dot{V}O_{2}$ max should be and how this should be statistically tested (Howley et al., 1995; Robergs et al., 2010; Niemeyer et al., 2021), whether a $\dot{V}O_{2}$ plateau is necessary in detecting $\dot{V}O_{2}$ max (Howley, 2007; Niemeyer et al., 2021; Noakes, 1997; 2008; Poole and Jones, 2017; Robergs et al., 2010), if secondary criteria have adequate test sensitivity and specificity for verifying $\dot{V}O_{2}$ max in the absence of a $\dot{V}O_{2}$ plateau (Howley et al., 1995; Poole and Jones, 2017; Wagner et al., 2020), or whether there is a need for a subsequent constant load non-steady state bout to verify whether peak $\dot{V}O_{2}$ is $\dot{V}O_{2}$ max (Astorino et al., 2009; Costa et al., 2021; Midgley and Carroll, 2009; Poole and Jones, 2017; Niemeyer et al., 2021). The research evidence that has contributed to many of these topics will be addressed in Parts-2 (the transition to continuous incremental exercise protocols and rapid response electronic gas analysers) and -3 (contemporary issues) of this series.

## Use of discontinuous incremental exercise to measure $\dot{V}O_{2}$ max and the $\dot{V}O_{2}$ plateau: 1920 to 1961

The topics of $\dot{V}O_{2}$ max and the $\dot{V}O_{2}$ plateau at $\dot{V}O_{2}$ max have been combined because historically, and physiologically, they were detected simultaneously and, as such, were initially defined by each other. However, to begin this topic, it is essential to focus on the measure of $\dot{V}O_{2}$ max, and during this process, comments will be directed to how data were used to identify (rightly or wrongly) the presence of a $\dot{V}O_{2}$ plateau.

### Research to 1924

As previously stated, Hill and Lupton (1923) have been repeatedly attributed as the scientists who first documented and defined the measure of $\dot{V}O_{2}$ max (Bassett, 2002; Hawkins et al., 2007; Howley, 2007; Millet et al., 2023). However, when reading the research of Hill and Lupton (1923) and Hill et al. (1924a), Hill et al. (1924b), Hill et al. (1924c), it is clear that numerous scientists had presented data on the measurement of $\dot{V}O_{2}$ max as early as 1913 (see below). Such results had informed Hill and Lupton of the methods for their research and subsequent data interpretations. This is best seen in their writing, where they commented, “*Very many observations have been made by physiologists of the maximum oxygen intake in man, and in the following table we give a selection of the highest values*” (Hill et al., 1924c). These $\dot{V}O_{2}$ max results spanned four prior publications and revealed data on $\dot{V}O_{2}$ max spanning 2,080 to 3,750 mL·min^−1^ for exercise modes of cycling, swimming, climbing, pushing a motorcycle, running, skiing and skating (Hill and Lupton, 1923, p. 153). These $\dot{V}O_{2}$ max values were lower than Hill and Lupton’s data on Hill of 4,175 mL·min^−1^ while running to exhaustion on an 84.5 m circular grass track at 267 m·min^−1^ (Hill and Lupton, 1923, p. 153–154).

Evidence of prior research on $\dot{V}O_{2}$ max is also seen in Hill and Lupton’s correction of past interpretations on the measure of $\dot{V}O_{2}$ during intense exercise and the attainment of $\dot{V}O_{2}$ max at exhaustion. Such interpretations were of these author’s proposed increasing efficiency of running at higher speeds (decreased Δ $\dot{V}O_{2}$ /Δ running speeds from 140 to 300 m·min^−1^), which Hill and Lupton corrected by stating, “*The explanation is simple: the participants of their experiments were not in a genuine steady state at the higher speeds …. it is clear that the maximum oxygen uptake of 3.3 L per min. was attained at a speed of 186 m per min. Hence, however fast N.S. ran above this speed he did not use more oxygen, not because he did not require it, but because he could not get it.*” (Hill and Lupton, 1923, p. 151). Of course, today we can correct Hill and Lupton’s explanation by replacing the “*could not get it*” with ‘was not able to consume it at a higher rate’ and thereby incorporate an awareness of both central and peripheral determinants of VO_2_max and the VO_2_ plateau. Though for all we know, this could have been their intended interpretation.

**FIGURE 1:**
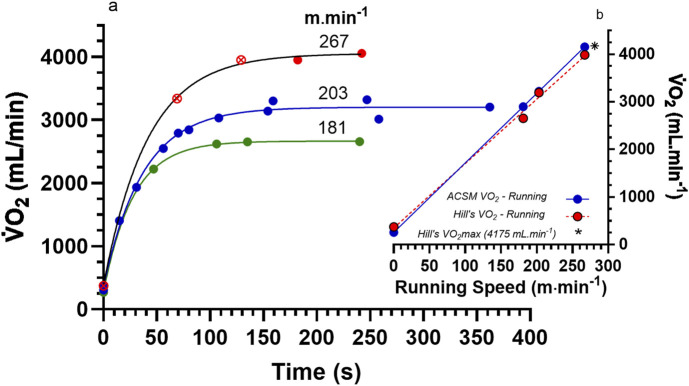
A reproduction of the original data from Hill and Lupton (1923) for their classic research on the $\dot{V}O_{2}$ response to different running speeds as presented in their Fig. 2 (p. 149) and expanded for the complete data of the 203 m·min^−1^ condition from results of their Table III (p. 150). **(a)** The original three bouts of level over-ground running (181, 203, and 267 m·min^−1^) where steady state exercise was attained for the speeds of 181 and 203 m·min^−1^. Note the red circle symbols (•) for the final two data points of the 267 m·min^−1^ non-steady state exercise bout. **(b)** The resultant linear regression for running speed vs. $\dot{V}O_{2}$ for treadmill running from the American College of Sports Medicine (Riebe et al., 2018, Table 6.3, p.152), and the final $\dot{V}O_{2}$ values of Hill for the speeds of 181 and 203 m·min^−1^. Hill was the participant in this data and weighed 73 kg. See the text for further explanations. (*Hill’s $\dot{V}O_{2}$ max = 4,175 mL·min^−1^).

Hill and Lupton (1923) developed their data interpretations of the concept of $\dot{V}O_{2}$ max from the results of five participants, although they presented the complete data for only one (A.V. Hill). Moreover, they only commented on this single data set (no evidence from the other four participants was presented in any form) in defining $\dot{V}O_{2}$ max and illustrating the levelling in $\dot{V}O_{2}$ during both steady state and non-steady state exercise. Such data (Figure 1a) were obtained by Hill sampling his own expired air while running on an 84.5 m circular grass track at different running speeds (181, 203, and 267 m·min^−1^) with assistance provided by the verbal feedback of the time for each lap. Hill was a highly trained, though non-elite distance runner. Expired air was sampled in a Douglas bag for 30 s at varied time intervals ranging from approximately 30 s for the initial 2 min, to 0.75–1 min after 3 min of exercise. For example, the initial bout of each of the two 203 m·min^−1^ exercise condition involved a 30 s time sample from 45 to 85 s with a 70 s central time stamp representing the central time of the sample interval. For the initial bout of the repeated condition, expired air sampling spanned 0–30 s, with a 15 s central time stamp (see Hill and Lupton, 1923; Table III, p. 150). Between the bouts of a specific running intensity, Hill stopped and recovered for 10–12 min and then ran the second bout at the same running speed but sampled expired air at a later time period. This process continued, with repeated bouts at the same pace through to 4–6 min. Such testing was continued through running velocities equal to 181 m·min^−1^, 203 m·min^−1^, which was completed twice with slightly different times of expired air sampling, and again for the final velocity equal to 267 m·min^−1^. This highest running velocity caused Hill to reach volitional exhaustion in just over 4 min. Plotting the entire $\dot{V}O_{2}$ responses (across multiple bouts of the same running velocity) revealed the levelling-off of the $\dot{V}O_{2}$ response when near exhaustion that coincided with and defined their definition of $\dot{V}O_{2}$ max (Figure 1a, 267 m·min^−1^ condition, • data points = purported levelling-off) at 4,175 mL·min^−1^.

When the peak $\dot{V}O_{2}$ responses for each running velocity are plotted against increasing exercise intensities, there is a linear increase in $\dot{V}O_{2}$ (Figure 1b), with no depiction of a plateau response. The measured $\dot{V}O_{2}$ data from 1920’s instrumentation and methodology (Figures 1a, b) are remarkably similar to that of steady state $\dot{V}O_{2}$ prediction from the ACSM equation for treadmill running (Liguori et al., 2022). Hill’s $\dot{V}O_{2}$ max is also similar to predicted values from the ACSM equation.

**FIGURE 2:**
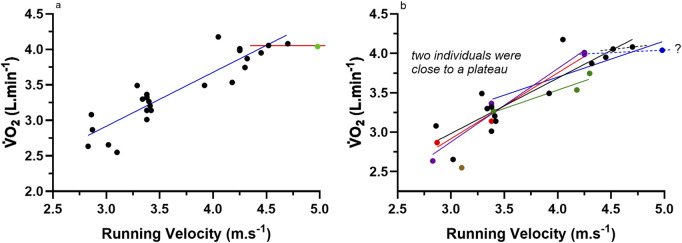
Data from Hill et al. (1924c); Table 1, p. 156, and Figure 1, p. 157 for the $\dot{V}O_{2}$ from multiple participants during discontinuous incremental exercise testing while running at different velocities on level ground. **(a)** All data without individual participant identification. The solid blue line is the linear regression of the data without the final three data points. The dashed line with a slope = 0 was manually applied. The green single data point (•) is identified as the one that gives the appearance of a $\dot{V}O_{2}$ plateau response. **(b)** All data from 7 participants with symbols for Hill’s data (•) identified (n = 14 data points), with different colours for the other participants. Participants 2 - 7 had 3 data points per participant, with participants 6 and 7 only having 1 data point each. Individual participant linear regression lines are shown for 5 of the participants, and the final two data points of two subjects identified by the black (from Hill) and blue dashed lines, reveal individual evidence from two subjects for a trend consistent with the levelling of the VO_2_ response.

Based on the data presented, Hill’s data does not support the concept of a $\dot{V}O_{2}$ plateau at $\dot{V}O_{2}$ max. Noakes (2008) has also expressed a similar interpretation, though without the clarity of the data presentation of Figures 1a,b, 2a,b. Presumably, Hill and Lupton were unaware of the limitations of their discontinuous multiple bout protocol, which induced the mono-exponential increase in $\dot{V}O_{2}$ . The levelling-off in $\dot{V}O_{2}$ during each constant intensity bout was caused by how an increase in exercise time progressively decreased the remaining Δ $\dot{V}O_{2}$ needed to reach the $\dot{V}O_{2}$ demand of the running velocity, further complicated by the cardio-respiratory and muscular endurance capacities of Hill. In current time, such knowledge and data interpretation are rudimentary to the understanding of the $\dot{V}O_{2}$ response and related accumulating oxygen deficit to exercise transitions to an increased steady state or moderate non-steady state (O’Malley et al., 2024).

In 1924 Hill and Lupton reproduced and explained their research from 1923 in an altered and more detailed presentation of their prior data, in addition to other data on similar and additional topics. Such evidence and explanation were presented across eight parts in three different publications by the Royal Society; Parts I-III (Hill et al., 1924a), Parts IV-VI (Hill et al., 1924b), and Parts VII-VIII (Hill et al., 1924c). The publication and content of Parts VII-VIII are the most pertinent to this review. Within this publication, Hill et al. (1924c) presented $\dot{V}O_{2}$ data from multiple participants (n = 7, Table 1, p. 156) for discontinuous over-ground running exercise at different velocities. Hill’s own data formed most of the data points and thereby was dominant in the $\dot{V}O_{2}$ -running velocity relationship. Such data are presented in Figure 2a and reveal a clear $\dot{V}O_{2}$ plateau response. Nevertheless, as the plateau response is only apparent for the last two data points, simply removing the last data point would remove the evidence of a plateau response. Nevertheless, this profile of $\dot{V}O_{2}$ to exercise intensity is still seen today in many textbooks of exercise physiology (Kenney et al., 2021; McArdle et al., 2023), yet as discussed below, such a data set and graphical presentation may be misleading.

The data of Figure 2a should have received, and continue to receive, more scrutiny. For example, which data points are from which participants? Hill’s data points are identified in Figure 2b, as are the individual regression lines of the data from the participants within the 7 that had more than 3 data points. As shown, two participants show evidence of data that clearly deviate from the $\dot{V}O_{2}$ gain response of the prior data points. These subjects are the data from Hill (black dots) and another subject (blue dots). Such trends towards the levelling of the $\dot{V}O_{2}$ response is documented by the dashed black and blue lines, respectively. This historic data clearly reveals the potential error in combining data from different participants to decipher a physiological response of individual participants. Consequently, such data are misleading and far from the convincing evidence of a $\dot{V}O_{2}$ plateau at $\dot{V}O_{2}$ max that has been promoted by the historical narrative of Hill’s work since this time (Bassett, 2002; Millet et al., 2023). As Hill and Lupton (1923) and Hill et al. (1924c) did not identify these individual subject responses as evidence for their proposal of a levelling off in $\dot{V}O_{2}$ at $\dot{V}O_{2}$ max, they can be given credit for conceptualizing the concept, but not proving it. In science, the two issues are very different, with the latter recognized as far more important.

### Where is the theory?

Before there can be further progress in this review, it is important to note that when reviewing the results of Hill and Lupton (1923), there was no evidence for the development of a theory. The researchers before and including Hill and Lupton measured the maximal rate of $\dot{V}O_{2}$ , and while Hill and Lupton provided a definition, science requires that a theory be developed so that other researchers can devise hypotheses to either further refine the theory, or critically challenge it. This deficiency is not so much a criticism of these pioneering researchers as it is a critical reflection on the research that followed. As we now know today from a more detailed understanding of the scientific method, it is premature to accept observations and interpretations as facts when they have not been critically challenged and/or replicated numerous times by other scientists. A suitable theory of $\dot{V}O_{2}$ max in the historical and methodological context present in 1923 would be as follows;

Theory 1a
*Multiple Bouts Of Intense Exercise That Successively Increase In Intensity Reveal A Levelling In $\dot{V}O_{2}$ , Where The Highest Value Not Surpassed By Further Exercise Is $\dot{V}O_{2}$ max.: 1924*

*During 2–6 min of constant intensity running exercise, $\dot{V}O_{2}$ increases in a non-linear fashion, eventually reaching a steady value that cannot be surpassed despite continued effort, even if it ends in exhaustion. This levelling, or plateau response in $\dot{V}O_{2}$ , reveals the peak value of $\dot{V}O_{2}$ that can be sustained for that exercise mode and intensity. Repeating this task for higher exercise intensities will allow the graphing of the peak $\dot{V}O_{2}$ responses across all exercise intensities, identifying the levelling in $\dot{V}O_{2}$ and as such the highest $\dot{V}O_{2}$ that can be attained by an individual; the individual’s maximal oxygen consumption ( $\dot{V}O_{2}$ max).*

Theory 1a is important in revealing six features that can be challenged by further research; 1. the discontinuous and multiple exercise bout feature of the incremental protocol used, 2. exercise mode specificity, 3. the lack of clarity in how many exercise bouts are required, 4. at what exercise intensities, 5. how the data should be presented, and 6. what criteria should be used to establish a $\dot{V}O_{2}$ levelling-off, or plateau, response? This last item is vitally important, for it once again needs to be stressed that Hill and Lupton did not appropriately present evidence of a levelling-off in peak $\dot{V}O_{2}$ responses from multiple bouts of constant intensity exercise that collectively spanned a range of intensities. As has been stated prior and further clarified below, Hill and Lupton (1923) just accepted the highest $\dot{V}O_{2}$ data point from their highest constant intensity bout that ended in volitional exhaustion as $\dot{V}O_{2}$ max. It is plausible they were confused by the graphical depiction of their constant intensity running bouts. Nevertheless, the added data from their more detailed presentation of their results (Hill et al., 1924c) resulted in their infamous, though incorrect (it reveals an opinion (non-evidence based summation) of a predominant cardio-pulmonary limitation to VO_2_max and the cause of the VO_2_ plateau), interpretation and explanation of the plateau at $\dot{V}O_{2}$ max as, “*The oxygen intake attains its maximum value, which in athletic individuals of about 73 kg body-weight is strikingly constant (in the case of running) at about 4 L per minute. The oxygen intake fails to exceed this value, not because more oxygen is not required, but because the limiting capacity of the circulatory-respiratory system has been attained.*” (Hill et al., 1924c, p. 157). The statement is also incorrect due to the fact that only 2 of 7 subjects demonstrated a $\dot{V}O_{2}$ response that may have been supportive (though not quantified by an objective criterion) of the interpretation.

In 1924, Hill et al. (1924c) further proposed that the cause of a $\dot{V}O_{2}$ plateau at $\dot{V}O_{2}$ max was likely to be due to “*…limitations of the circulatory respiratory system.*” (p. 166). There was an opportunity here to word another theory concerning the legitimacy of this opinion, however, as this topic has received considerable commentary and research in recent times, and as it is the purpose of this review to focus on methodological features of the $\dot{V}O_{2}$ responses to incremental exercise, we direct the reader to previously published work on the evidence for and against cardiopulmonary limitations to incremental exercise (Bassett and Howley, 1997; 2000; Noakes, 1997; 1998; 2011; Levine, 2008; Shephard, 2009; Ferretti, 2014; Lundby et al., 2017; Hoppeler, 2018; Joyner and Dominelli, 2021; Wagner, 2023).

The research of Hill and Lupton (1923) and Hill et al. (1924c) was not perfect. While some of their laboratory methods remain sophisticated and accurate (e.g., chemical determination of gas fractions in expired air), their exercise methodology was simplistic, and they proposed the concept of $\dot{V}O_{2}$ max in 1923 based on one running velocity which caused Hill to reach a stable $\dot{V}O_{2}$ across 2–3 min and immediately before volitional exhaustion. Such research also included a clear cardio-pulmonary bias to their understanding of the cause of the $\dot{V}O_{2}$ plateau, and where this interpretation was based on opinion and not experimental research acquired evidence. The research of the importance of cardiovascular (central and peripheral components), pulmonary and peripheral gas exchange, neuromuscular (central and peripheral components) and additional peripheral physiological and biochemical determinants of $\dot{V}O_{2}$ max and the $\dot{V}O_{2}$ plateau will be presented in parts-2 and -3 based on the time-periods within which this research was published.

To their credit, the data presented by Hill et al. (1924c) did provide evidence in two subjects for the likely presence of the $\dot{V}O_{2}$ plateau at $\dot{V}O_{2}$ max. Unfortunately, it was the interpretation that such a $\dot{V}O_{2}$ plateau response should occur in all participants that was ill-founded. In all fairness, it is wrong to have expected more. Such research-based expansion and clarification of Hill’s research and scholarship were a challenge for other researchers in the years that followed. A review of this work to 1961 is presented next.

### Research after 1924

Robinson (1938) studied the physiological responses at rest and during exercise and recovery for 93 male volunteers aged 6–91 years. No additional methodological details were provided of the exercise conditions or details used to decipher $\dot{V}O_{2}$ max. The $\dot{V}O_{2}$ max of the participants was highest for participants in their late teens to mid-30 years, varying from 4.0 to 4.5 L·min^−1^ (53–63 mL·kg^−1^·min^−1^). While the $\dot{V}O_{2}$ max data across ages were interesting, the limited explanation of the methods used to measure $\dot{V}O_{2}$ max renders this research of limited value to this review.

Taylor et al. (1955) thoroughly investigated the $\dot{V}O_{2}$ response to discontinuous incremental treadmill running exercise testing. Due to the importance of this research to future research methodology concerning the $\dot{V}O_{2}$ plateau at $\dot{V}O_{2}$ max for multiple decades that followed, it is necessary to detail the methods of this research. The participants of the Taylor et al. (1955) study were 115 males between the ages of 18–35 years, and all were medically evaluated to ensure good health. Participants varied in fitness from collegiate-level distance runners to minimal recreational activity. Prior to discontinuous incremental exercise testing, participants first completed an endurance fitness test on the treadmill (modified Harvard Fitness [Step] Test). However, the details of the treadmill modification of the test were not provided. The results from this initial test determined the initial %grade used on the treadmill for running at 7 mi·hr^−1^ (11.2 km·hr^−1^). Such start conditions were typically 5 or 7.5 %grade, and the test commenced 5 min after a long (5–60 min) warm-up of walking at 3.5 mi·hr^−1^ (5.6 km·hr^−1^) at a 10 %grade.

During the testing of $\dot{V}O_{2}$ max, the different bouts of exercise were performed on different visits to the laboratory (assumed to mean different days). Participants ran on the treadmill for 3 min at the desired %grade and speed. Expired air was collected for 1 min starting at 1.75 min and analysed for expired gas fractions and ventilation rate for computations of indirect calorimetry. For the next test, the %grade was increased by 2.5% and the procedures were repeated. Such testing conditions were repeated until participants reached volitional exhaustion within this time frame and where further discontinuous incremental testing revealed consecutive $\dot{V}O_{2}$ values that varied by < 150 mL·min^−1^ or 2.1 mL·kg^−1^·min^−1^. Such criteria were accepted to reveal a $\dot{V}O_{2}$ plateau and where the highest $\dot{V}O_{2}$ was defined as $\dot{V}O_{2}$ max. Taylor et al. (1955) purposedly verified the validity of their methods to measure $\dot{V}O_{2}$ max and the $\dot{V}O_{2}$ plateau by documenting stable $\dot{V}O_{2}$ data across expired air collections from 1.75 to 2.75 min vs. 2.75–3.75 min (3.45 vs. 3.48 L·min^−1^, respectively) in 10 other participants. This was done to document their assumption that a peak $\dot{V}O_{2}$ can be attained within 3 min (the duration of each bout of exercise) for steady and non-steady state exercise.

**FIGURE 3:**
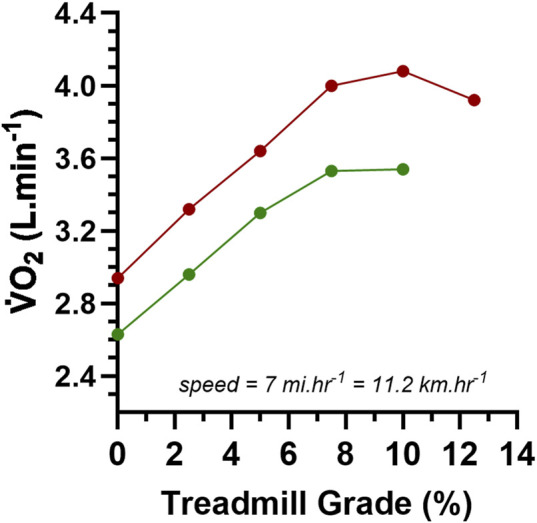
Data from a Taylor et al. (1955) for $\dot{V}O_{2}$ data for two different participants during treadmill exercise at 7 mi. hr^−1^ at different inclines (%grade) to volitional exhaustion.

The $\dot{V}O_{2}$ plateau criterion was based on the measured increase in $\dot{V}O_{2}$ for a 2.5 %grade increment at 7 mi·hr^−1^, which Taylor et al. (1955) measured during additional testing of 13 participants (details not provided) across three additional exercise intensities below the participant-specific $\dot{V}O_{2}$ max. The mean $\dot{V}O_{2}$ increment was 299.3 ± 86.5 mL·min^−1^ (4.18 ± 1.07 mL·kg^−1^·min^−1^; this reveals the participants’ mean body mass = 71.6 kg). Taylor et al. (1955) simply halved this value and recommended <150 mL·min^−1^ to be their $\dot{V}O_{2}$ plateau at $\dot{V}O_{2}$ max criterion. Representative data from two participants are presented in Figure 3, and such data are interesting because they show two examples of an apparent $\dot{V}O_{2}$ plateau and $\dot{V}O_{2}$ max. Given that this study used 115 participants of varied fitness, it would have been interesting to know the data for the participants who did or did not meet the $\dot{V}O_{2}$ plateau criterion. Unfortunately, Taylor et al. did not provide these results, which, given the data examples of Figure 3, indirectly allowed the concept of a $\dot{V}O_{2}$ plateau at $\dot{V}O_{2}$ max as presented by Hill and Lupton (1923) and Hill et al. (1924c) to be further engrained into the rapidly growing and accepted epistemology of exercise physiology. This of course means that as of 1955, there was still no research-acquired data documenting a VO_2_ plateau response at VO_2_max in the majority of a reasonably large sample of human participants.

The ACSM equation for treadmill running for a 71.6 kg person computes the $\dot{V}O_{2}$ cost for an increment of 2.5 %grade at 7 mi·hr^−1^ at 302 mL·min^−1^ (4.22 mL·kg^−1^·min^−1^), thereby revealing the accuracy of the methods and resultant data from this early research. However, the $\dot{V}O_{2}$ increment for a 2.5 %grade is treadmill belt speed dependent; the higher the treadmill belt speed, the larger the $\dot{V}O_{2}$ demand for a 2.5 %grade increment (Robergs, 2007). As such, it would be invalid to apply this criterion to exercise conditions different to treadmill exercise involving stage increments of 2.5 %grade at 7 mi·hr^−1^. This is a limitation of the generalisability of the absolute expression of the Taylor et al. (1955) $\dot{V}O_{2}$ plateau at $\dot{V}O_{2}$ max criterion. Ironically, the relative expression of the criterion, that of the $\dot{V}O_{2}$ plateau being present if the change in $\dot{V}O_{2}$ was <50% of the $\dot{V}O_{2}$ demand of the stage (or ramp) increment, would have been the feature to apply to future research (see Part-2 of this review series).

The importance of the Taylor et al. (1955) $\dot{V}O_{2}$ plateau criterion cannot be over-stated. As will be detailed in Part-2 of this series, this criterion has been used repeatedly in research since 1961, with documented application in research as current as 2006 (Kropej et al., 2005; Midgley et al., 2007), though likely to have even more recent use.

### A revised theory of $\dot{V}O_{2}$ max and the $\dot{V}O_{2}$ plateau at $\dot{V}O_{2}$ max

Given the repeated use of the Taylor et al. (1955) protocol in studies within and beyond the time period of this review, it is pertinent to revise Theory 1a to accommodate the features of the protocol and $\dot{V}O_{2}$ plateau definition.

Theory 1b
*Multiple Bouts Of Intense Exercise That Successively Increase In Intensity Reveal A Levelling In $\dot{V}O_{2}$ , Where The Highest Value Not Surpassed By Further Exercise Is $\dot{V}O_{2}$ max.: 1955*

*During constant running at any intensity, $\dot{V}O_{2}$ increases non-linearly to a steady value within a 3 min duration. Repeated administration of a higher exercise intensity in a subsequent 3 min bout after 10 min or more of rest will cause a higher steady $\dot{V}O_{2}$ value to be attained. Repeated bouts of a higher exercise intensity eventually cause $\dot{V}O_{2}$ responses that cannot be increased more than 150 mL·min^−1^. This levelling, or plateau response in repeated peak $\dot{V}O_{2}$ efforts reveals the maximum rate of $\dot{V}O_{2}$ that can be sustained for that exercise mode ( $\dot{V}O_{2}$ max).*

Theory 1b has three aspects that can be assessed through further research; 1. The attainment of steady $\dot{V}O_{2}$ values within 3 min, regardless of the absolute intensity and relative effort, for both steady state and non-steady state exercise intensities. 2. The use of 10 min rest intervals does not cause carry-over effects to subsequent additional 3 min bouts of increasing intensity. 3. Using repeated 3 min bouts of a higher exercise intensity that eventually cause $\dot{V}O_{2}$ responses that cannot be increased by more than 150 mL·min^−1^ (if exercise was continued beyond 3 min the $\dot{V}O_{2}$ would not increase my more than 150 mL·min^−1^) is a valid criterion for defining the presence of a $\dot{V}O_{2}$ plateau.

Early evidence of critical inquiry of Hill and Lupton’s original research was presented by Mitchell et al. (1958), though such critical reflection was directed to the central cardiovascular interpretations of the data and not the features of the exercise protocols or the related VO_2_ data processing. For example, within the Introduction, Mitchell et al. (1958) stated, “*The difficulty, insofar as maximal oxygen intake is concerned, is simply that its physiological meaning is imperfectly understood. The view that cardiac capacity is determinant of maximal oxygen intake is surmise, not established fact.*” (Mitchell et al., 1958, p. 538). Mitchell et al. (1958) measured the $\dot{V}O_{2}$ response discontinuous incremental exercise in 65 men using a similar protocol to Taylor et al. (1955) except that the treadmill speed was 6 mi·hr^−1^ and all testing was completed on the 1 day with 10 min rest separating each 3 min exercise bout. They reported that for most participants testing was completed within 1.5 h. As steady-state $\dot{V}O_{2}$ rose 142 mL·min^−1^ with each 2.5% increase in treadmill grade at 6 mi·hr^−1^, the authors adopted a much more stringent criterion for the $\dot{V}O_{2}$ plateau at <54 mL·min^−1^ for consecutive test bouts ending in volitional exhaustion. This criterion was attained in 72% of the participants. The ACSM equation for treadmill running computes the $\dot{V}O_{2}$ cost for an increment of 2.5 %grade at 6 mi·hr^−1^ at 3.62 mL·kg^−1^·min^−1^. Mitchell et al. (1958) did not report the descriptive characteristics of the participants or provide adequate details for how they obtained their $\dot{V}O_{2}$ increment values. However, for a 75 kg person, the ACSM calculated $\dot{V}O_{2}$ increment equals 271.5 mL·min^−1^. Consequently, the $\dot{V}O_{2}$ increment data of Mitchell et al. (1958) (142 mL·min^−1^) is non-physiologically low and, as such, flawed, and the subsequent $\dot{V}O_{2}$ plateau criterion they used should be disregarded.

Despite the poor validity of some of the measurements in the Mitchell et al. (1958) study, this was the first research and commentary to reveal the dilemma of how the protocol-dependent increase in $\dot{V}O_{2}$ influences the construct validity of any change in consecutive $\dot{V}O_{2}$ data points used to define the $\dot{V}O_{2}$ plateau. As previously mentioned, this dilemma has plagued research on this topic to the present time.

Wyndham et al. (1959) published a detailed study of repeated discontinuous incremental exercise testing using four male participants. The rationale for the research was based on the absence of data from Hill and Lupton (1923), Hill et al. (1924c), Taylor et al. (1955) and Mitchell et al. (1958) for profiling the actual $\dot{V}O_{2}$ responses at the top end of the $\dot{V}O_{2}$ to exercise intensity curve leading to the proposed levelling off or what was re-expressed as the $\dot{V}O_{2}$ plateau. As such, Wyndham et al. (1959) viewed the work of the prior authors’ acceptance of the $\dot{V}O_{2}$ plateau at $\dot{V}O_{2}$ max as more supposition than fact. For example, what were the final kinetics of the non-linear increase in $\dot{V}O_{2}$ for different discontinuous exercise intensities leading to a $\dot{V}O_{2}$ plateau response? Is there a way to mathematically profile this response? This was revolutionary research inquiry for this time-period, and further indicative of advanced thinking and the need for evidence-based data interpretation before escalating supposition (opinion) to fact for the presence of a $\dot{V}O_{2}$ plateau response at $\dot{V}O_{2}$ max.

The four participants were exercise trained using cycle ergometry for 4 months prior to data collection to diminish any training-induced carry-over effects for improvement to $\dot{V}O_{2}$ max during the time-course of the research. The training also enabled the participants to be able to complete the rigorous exercise testing involved in the research. Nevertheless, each participant was only moderately trained with their highest exercise intensity conditions (not necessarily at or close to $\dot{V}O_{2}$ max) being 271, 225, 260 and 282 W for participants a, b, c and d, respectively. Each participant completed between four to eight repeated bouts of a given intensity, with between eight to 11 different intensities studied depending on the participant (56, 113, 158, 169, 181, 192, 203, 215, 225, 237, 249, 260, 271, 282 W). Thus, each participant completed, on average, 54 different constant load exercise bouts, yielding not only quality data for assessing the non-linear profiling of the $\dot{V}O_{2}$ responses, but also data for test-retest reliability and variability (error). Data for heart rate were also obtained to test the assumption from prior research for the ability to linearly extrapolate exercise heart rates to estimated $\dot{V}O_{2}$ max (though these heart rate data are not pertinent to this review). Unfortunately, added methodological details of each of the exercise bouts were not provided, other than for the more intense exercise bouts, where expired gas sampling occurred across 1 min, but for lower exercise intensities, this time frame had to be increased up to 3 min. Expired gas fractions were measured using the Haldane chemical gas analysis method.

**FIGURE 4:**
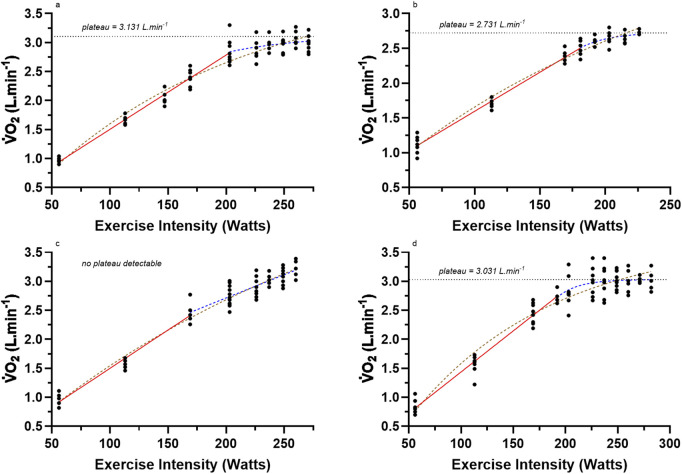
Reproduced data from the four participants **(a–d)** from Wyndham et al. (1959) who were meticulously studied to assess their peak $\dot{V}O_{2}$ responses for repeated bouts of constant intensity cycle ergometer exercise at multiple intensities spanning low to very intense exercise (see text).

Results from the test-retest assessment of each exercise bout were also not presented or discussed, other than the tabled data for the standard error of the mean for the three highest repeated $\dot{V}O_{2}$ max data points. Consequently, the standard error of the mean was converted to the root mean square error for each participant (0.081, 0.087, 0.108, 0.099 L·min^−1^, respectively) and expressed relative to their peak $\dot{V}O_{2}$ response (2.7, 3.2, 3.59, 3.16%, respectively) with a mean relative error for $\dot{V}O_{2}$ max being 3.16%. Due to the importance of this research for being the first study to non-linear profile $\dot{V}O_{2}$ results progressing to a near levelling (plateau) for each exercise bout, the published individual data were retrieved as accurately as possible and presented in Figure 4 (data for exercise intensity were converted from ft-lb·min^−1^ to Watts; 1 W = 0.022597 ft-lb·min^−1^). The authors did not detail their nonlinear computational model for their data, but as shown in Figures 4a–d, it clearly was not a mono-exponential function. The $\dot{V}O_{2}$ data points for participant c are close to linear, though note the peak $\dot{V}O_{2}$ data reveal that this person had possibly the largest $\dot{V}O_{2}$ max value of the four participants. Without knowing the actual pulmonary gas exchange data and related variables, the linearity of the $\dot{V}O_{2}$ data is hard to interpret, with possible causes being an insufficiently high enough final intensity, or other features such as a large O_2_ cost of ventilation and/or a high slow twitch skeletal muscle expression of the quadriceps, gluteal and lower leg musculature. Nevertheless, for participants a, b, and d, the best fit of the data was a combined initial linear segment followed by a mono-exponential fit rising to a plateau, which is identified in the figures.

Interestingly, the initial linear fits for participants a, b, c, and d were 12.80, 11.22, 13.21, and 14.17 mL·min^−1^·Watt^−1^, respectively. This is interesting because such $\dot{V}O_{2}$ slope data for participants a, b, and c all align well with the known 11–13 mL·min^−1^·Watt^−1^ $\dot{V}O_{2}$ gain of exercise across repeated bouts of steady-state exercise (Barstow and Mole, 1991; Medbo, 1996). Based on the Taylor et al. (1955) $\dot{V}O_{2}$ plateau criteria, the $\dot{V}O_{2}$ max exercise intensity for these participants would have occurred at 237 W (2.97 L·min^−1^), 181 W (2.5 L·min^−1^), 226 W (2.87 L·min^−1^) and 203 W (2.83 L·min^−1^), respectively. For all participants, the delta $\dot{V}O_{2}$ between the actual peak $\dot{V}O_{2}$ data point and the Taylor criterion detected $\dot{V}O_{2}$ max (= underestimation error) were 192, 230, 350, and 180 mL·min^−1^, respectively (though note the complication in the different exercise modes of the two studies; treadmill running (Taylor et al.) vs. cycling Wyndham et al.)).

The data of Figure 4 are important for they show the limitations of the common approach for that era to assume that the peak $\dot{V}O_{2}$ attained without a further increase > 150 mL·min^−1^ for a higher exercise intensity was sufficient to accept as a $\dot{V}O_{2}$ plateau with the highest $\dot{V}O_{2}$ value recognized as $\dot{V}O_{2}$ max. The data show three critical features of the $\dot{V}O_{2}$ response to discontinuous incremental exercise protocols that, for this time period and available instrumentation, only mathematical modelling of the repeated data trials can reveal: 1. there is a deviation from linearity of the $\dot{V}O_{2}$ response to discontinuous exercise bouts of increasing intensities characterized by a decreasing $\dot{V}O_{2}$ increment for given increases in exercise intensity (decreasing $\dot{V}O_{2}$ gain), 2. there are individual differences in the exercise intensity where this deviation commences, and 3. such results support the development of a $\dot{V}O_{2}$ plateau response in some individuals. The data’s added disturbing feature is the large error of measurement for the repeated bouts of each exercise intensity. Added to this is the continued use of large time averages for $\dot{V}O_{2}$ data collection during the discontinuous exercise bouts. Such evidence begs the question of when is a $\dot{V}O_{2}$ plateau a plateau if you are using single data points for a given exercise intensity? As already explained, the Taylor criterion remains inadequate as a solution to this problem.

From the perspective of which research study first documented the presence of a levelling off in the $\dot{V}O_{2}$ response during discontinuous incremental exercise to $\dot{V}O_{2}$ max, it is clear that it is the study of Hill et al. (1924c). Yet, as previously explained, despite having the data, these authors did not report on the trends in the $\dot{V}O_{2}$ of their individual subjects. The data of Wyndham et al. (1959) was the first to reveal the non-linearity that can occur for the $\dot{V}O_{2}$ plateau response, and due to that, along with what can be a sustained small increase in $\dot{V}O_{2}$ with further increases in exercise intensity, the error in using the Taylor et al. $\dot{V}O_{2}$ plateau criterion. As such, this study also showed the individual variability in the changing $\dot{V}O_{2}$ gain of whole body (pulmonary gas exchange determined) $\dot{V}O_{2}$ during incremental exercise to contractile failure. The future content of this review, and that for Parts-2 and -3, will show how this research was largely overlooked in the decades that followed, with clear favouritism in using the Taylor et al. $\dot{V}O_{2}$ plateau criterion.

In 1961, Astrand and Saltin (1961a) investigated the $\dot{V}O_{2}$ , heart rate, ventilation, and blood lactate responses to different non-steady state exercise bouts. The core studies that informed Astrand’s and Saltin’s research were each of Robinson (1938), Taylor et al. (1955), and Mitchell et al. (1958). Interestingly, Hill’s prior research was not cited.

**FIGURE 5:**
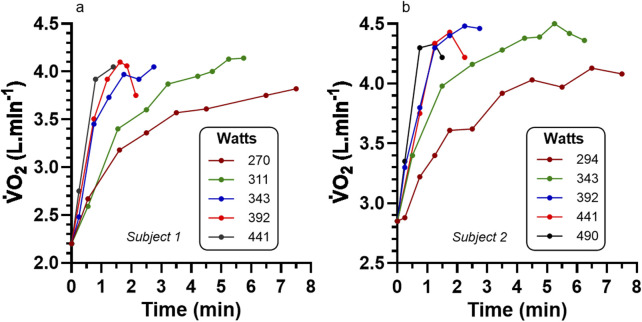
Data of $\dot{V}O_{2}$ from Astrand and Saltin (1961a) for cycle ergometry to volitional exhaustion for two **(a,b)** representative participants each completing 5 different exercise intensities. See Figure 6 for added coverage of pertinent $\dot{V}O_{2}$ peak data for all participants of this study.

Astrand and Saltin (1961a) required five moderately to highly ( $\dot{V}O_{2}$ max values ranged from 47 to 63 mL·kg^−1^·min^−1^) endurance-trained participants (1 female, 4 male) to complete multiple single bouts of constant load non-steady state intensity cycle ergometry at 50 rev·min^−1^. The $\dot{V}O_{2}$ data for two of the male participants (# 1, 2) are presented in Figure 5a, b. Participants first performed 10 min of moderate intensity cycling ( $\dot{V}O_{2}$ = 48–63% $\dot{V}O_{2}$ max), followed immediately by an increased intensity devised to cause volitional exhaustion in 2–8 min specific to each participant. During each bout, expired gas samples were collected into Douglas bags, and subsequently measured for volume (spirometer), and gas fractions were determined chemically by the Haldane technique. The exact timing of the expired air samples was not provided, though assessment of their graphed results revealed close to 30 s intervals for the initial 2 min, with 1 min intervals thereafter. The sample timing of Taylor et al. (1955) was referred to, implying that a 30 s sample time for an exercise time of 1 min involved gas sampling from 45 to 75 s, etc.

The targeted exercise intensities ranged from 196 to 294 W for the female participant (3 bouts), and 270 to 490 W for the four male participants (5, 5, 5, and 6 bouts). Time to volitional exhaustion ranged from 1.8 to 7.5 min. Details were not provided for whether tests were all conducted on different days, or if not, the length of recovery required between bouts. A total of 42 exercise bouts were performed across all participants, revealing that bouts were performed at least twice each to acquire sufficient data points per trial to profile the $\dot{V}O_{2}$ responses.

The results of Astrand and Saltin (1961a) are sufficient to refute the assumption inherent to the Hill and Lupton (1923), Hill et al. (1924c) and Taylor et al. (1955) protocols for the attainment of stable $\dot{V}O_{2}$ values within 3 min, regardless of the exercise intensity, so long as it was preceded by moderate intensity steady-state exercise.

The data also show that depending on the participant, there is a narrow range of exercise intensities where $\dot{V}O_{2}$ can increase to near-stable values within 3 min. More importantly, for the two representative participants shown for their study (Figures 5a, b), peak $\dot{V}O_{2}$ responses were highest for lower exercise intensities that required 5–6 min for the peak response. Unfortunately, the influence of the recovery duration between constant intensity bouts of exercise of different intensities (low to very intense) has received minimal research attention. Finally, the proposition and acceptance of a single absolute delta $\dot{V}O_{2}$ cut-off for verifying the attainment of a $\dot{V}O_{2}$ plateau is over-simplistic. As shown in Figure 5, a subject’s $\dot{V}O_{2}$ can continue to increase despite the slope of the $\dot{V}O_{2}$ response to constant intensity exercise being low. Criticism has already been expressed for the use of the Taylor et al. (1955) Δ $\dot{V}O_{2}$ cut-off value on the grounds of the protocol specificity of this value. Further criticism can be expressed regarding how such a $\dot{V}O_{2}$ plateau criterion should be more defined by the measure’s combined experimental error and biological test-retest variability. Such evidence is presented in Part-2 of this review series.

Astrand and Saltin (1961a) reported that statistical analyses revealed no differences in the peak $\dot{V}O_{2}$ values across the different discontinuous bouts. However, statistical analyses were not detailed, and with only five participants, with very different absolute exercise intensities, number of bouts, and bout durations, interpretations of non-significant results from statistics would logically be compromised due to low statistical power and an increased probability for type-II errors. When assessing the bouts that induced the highest peak $\dot{V}O_{2}$ values, the intensity and exercise duration data (in parentheses) for participants 1 to 5 were 294 W (6 min), 343 W (6.5 min), 319 W (6.5 min), 392 W (4.5 min), and 196 W (7.5 min), respectively. The peak $\dot{V}O_{2}$ responses of all participants were at the lower end of the exercise intensities of their multiple constant load bouts.

It is worth noting that Astrand and Saltin (1961a) used the term ‘ $\dot{V}O_{2}$ plateau’ to qualify the $\dot{V}O_{2}$ responses during individual constant load bouts and not the Watts vs. peak $\dot{V}O_{2}$ profile of the data. This is another example of the same error made by Hill and Lupton (1923) and Hill et al. (1924c) as detailed earlier. It is also important to mention that such an oversight in understanding and data interpretation on the topic of a $\dot{V}O_{2}$ plateau across both discontinuous and continuous exercise testing (to be presented in Part-2) has been repeated in research and textbook driven education for multiple decades ever since. This is unfortunate, as the data of Astrand and Saltin (1961a) and Wyndham et al. (1959) clearly document the progressively diminishing ability to increase peak $\dot{V}O_{2}$ with further increments in exercise intensities across repeated discontinuous bouts of exercise to contractile failure when testing moderately to highly endurance trained participants.

**FIGURE 6 F6:**
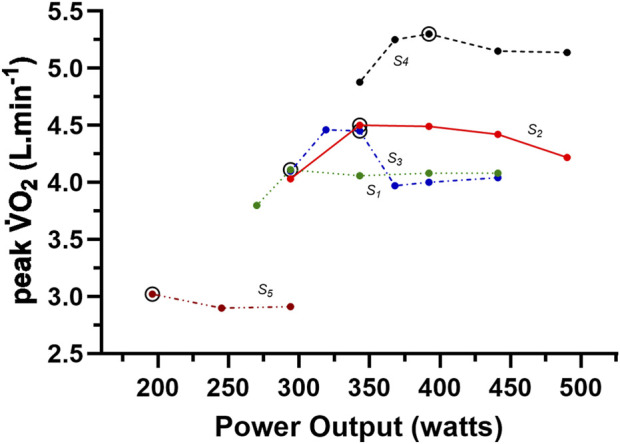
Data for relationship for Watts vs. peak $\dot{V}O_{2}$ for individual participants from Astrand and Saltin (1961a) who completed multiple bouts of cycle ergometry resulting in volitional exhaustion between 2 and 7.5 min in 5 participants. The large open circle for each participant designates their highest peak $\dot{V}O_{2}$ . Participants 1 to 4 (S_1-4_) demonstrated a $\dot{V}O_{2}$ plateau (see text) and hence m $\dot{V}O_{2}$ red $\dot{V}O_{2}$ max.

To document this feature, the data of Astrand and Saltin have been re-drawn as accurately as possible to reveal these individual participant relationships and presented in Figure 6. When comparing Figures 5a,b–6, [Fig F6], the consistent $\dot{V}O_{2}$ response to invoke the highest peak value ( $\dot{V}O_{2}$ max) required exercise intensities that incurred an exercise bout test time to volitional exhaustion approximating 4.5–7.5 min, or as mentioned prior, at the lower end of their exercise intensity range (bout durations longer than 3 min). For all but participant five, participants revealed a more considerable decrease in the peak $\dot{V}O_{2}$ response for longer rather than shorter test durations (lower vs. higher exercise intensities). However, it is clear from this data that the initial non-steady state exercise bout should incur volitional exhaustion in approximately 5 min. Interestingly, the data from participant five also reveal that for discontinuous exercise testing, a data point providing a lower $\dot{V}O_{2}$ before, as well as after the $\dot{V}O_{2}$ peak value, is needed to affirm the measure of $\dot{V}O_{2}$ max. The remaining concern over whether continuous incremental exercise is a more valid method than discontinuous protocols for inducing a $\dot{V}O_{2}$ max (causes higher $\dot{V}O_{2}$ max values) and a $\dot{V}O_{2}$ plateau is explored in Part-2 of this review series.

The results of Figures 5, 6 present a dubious view of more recent recommendations (Astorino et al., 2009; Costa et al., 2021; Midgley and Carroll, 2009; Niemeyer et al., 2021; Poole and Jones, 2017) for using a single verification bout at an intensity greater than that at $\dot{V}O_{2}$ max and causing volitional fatigue in 2–3 min. This topic will be addressed in Parts-2 and -3 of this review series. Furthermore, the results also show that even discontinuous exercise bouts involving non-steady exercise to volitional exhaustion are not equal in their likelihood to induce a $\dot{V}O_{2}$ max. Depending on the individual (genetically influenced motor unit proportion expression?), exercise bouts at intensities that are too short (2–3 min) will cause a too rapid onset of contractile failure (exhaustion) to attain $\dot{V}O_{2}$ max, while conversely, bouts with exercise intensities that are too low and cause exhaustion after 7 min do not provide sufficient $\dot{V}O_{2}$ demand to raise $\dot{V}O_{2}$ rapidly enough to reach $\dot{V}O_{2}$ max prior to the inevitable exhaustion that results from such longer duration accumulation of a $\dot{V}O_{2}$ deficit. As such, Astrand and Saltin (1961a) revealed that the discontinuous exercise protocol needs to have multiple stages to cater to individual variability in the ‘optimal’ stage, as well as to ensure that there is one stage on either side of the $\dot{V}O_{2}$ max stage that reveal lower, or negligible increase, in peak $\dot{V}O_{2}$ values to verify the validity of the measurement. Based on these findings, the data from Taylor et al. (1955) and Mitchell et al. (1958) would now be viewed as methodologically flawed.

### Refinement of the theory of a VO_2_ plateau at VO_2_max

We can now revise Theory 1b to improve the methodological inquiry linked to the measures of $\dot{V}O_{2}$ max and the $\dot{V}O_{2}$ plateau. This new version is presented below in Theory 1c, with new content underlined.

Theory 1c
*Multiple Bouts Of Intense Exercise That Sequentially Increase In Intensity Reveal A Levelling In $\dot{V}O_{2}$ , Where The Highest Value Not Surpassed By Further Exercise Is $\dot{V}O_{2}$ max.: 1961*

*During constant-intensity running or cycling exercise at an intensity sufficient to cause volitional exhaustion within a 4–7 min time period, $\dot{V}O_{2}$ increases in a non-linear fashion that may or may not eventually reach a steady value. Repeated administration of a higher exercise intensity in a subsequent bout after 10 min or more of rest will eventually cause $\dot{V}O_{2}$ responses that cannot be increased further than the prior maximal effort test that ended in exhaustion. This levelling, or plateau response in repeated peak $\dot{V}O_{2}$ efforts, reveals the maximum rate of $\dot{V}O_{2}$ that can be sustained for that exercise mode ( $\dot{V}O_{2}$ max). These responses are unique to each individual and there is no one relative non-steady state exercise intensity that induces $\dot{V}O_{2}$ max in all individuals.*

For this revised theory, three topics would require further research investigation: 1. the relative exercise intensities needed to induce a range of exercise time to exhaustion to cause the attainment of $\dot{V}O_{2}$ max, 2. the 10 min recovery duration between different constant load exercise bouts to failure, and 3. the ability of this methodology to reveal $\dot{V}O_{2}$ data showing at least one lower value for exercise intensities below and above the peak $\dot{V}O_{2}$ response (= $\dot{V}O_{2}$ max).

Interestingly, none of these research needs were adequately addressed in the following years. In large part, as explained in Part-2 of this review series, this was due to the rapid transition to continuous incremental exercise testing in combination with the redirected interest from $\dot{V}O_{2}$ max measurement to that of blood lactate and ventilatory measures of threshold changes in the metabolic response to incremental exercise. Furthermore, and as raised earlier, this early research of discontinuous incremental exercise testing has relevance to the topic of added bouts of intense, constant load exercise to verify $\dot{V}O_{2}$ max from prior exercise bouts or incremental exercise.

## Summary and conclusion

The time-period from 1920 to 1961 was fundamental to the foundational knowledge base of exercise physiology to the current time. However, a foundation built from errors in the pursuit and reinforcement of the related epistemology of a discipline can negatively impact the discipline in the years, decades, and perhaps the century that follows. The commonality of numerous errors in the human pursuit of science was a stark message from the work of Kuhn (1962) in his assessment of the historical development of the physical sciences, from which it can be assumed has relevance to all disciplines of science. Of even greater concern was Kuhn’s definition of the mainstream function of science seen through the historical development of the physical sciences, which, as shown below, was labelled by Kuhn as ‘normal science’.

Normal science’ was/is an attempt to *“… force nature into the preformed and relatively inflexible box that the paradigm supplies. No part of the aim of normal science is to call forth new forms of phenomena; indeed those that do not fit the box are often not seen at all. Nor do scientists normally aim to invent new theories, and they are often intolerant of those invented by others.”* (Kuhn, 1962, p. 24).

Where possible, science should seek to detect errors, correct or at least minimize them, and not function to reinforce them through the indifference to being far from the truth simply because it is ‘normal’, which is to say it is conventional, or safe. These words may seem harsh, but Kuhn himself directed them based on the prior definition, and Popper re-expressed them in even more emotive negative words (Popper, 1995). Indeed, the need to detect errors, and succeeding in doing this, provides a powerful directive to improve the understanding and application of this knowledge. This in turn, minimizes the harm done by repeated replication of error(s) to the discipline(s) at question.

The reality revealed from this review is that the initial description of the $\dot{V}O_{2}$ plateau at $\dot{V}O_{2}$ max was an error in data interpretation by Hill and Lupton (1923) and Hill et al. (1924c). No part of their research methodology described in their initial manuscript (1923) included a higher intensity exercise bout to verify that a peak $\dot{V}O_{2}$ response during a discontinuous exercise bout ending in exhaustion was indeed a maximal $\dot{V}O_{2}$ response to exercise. Conversely, their subsequent more detailed presentation of their data Hill et al. (1924c) revealed a plateau-like response in two of seven subjects, though as previously explained, such data are far from convincing of the expectation of a VO_2_ plateau in all subjects at VO_2_max. Interestingly, Taylor et al. (1955) did incorporate an added higher intensity exercise stage to their protocol, but their liberal definition of a $\dot{V}O_{2}$ plateau at half the $\dot{V}O_{2}$ demand increment of the next exercise bout (<150 mL·min^−1^), followed by their failure to present any data on the participants that did or did not meet the $\dot{V}O_{2}$ plateau criterion, gave indirect support of the $\dot{V}O_{2}$ plateau and $\dot{V}O_{2}$ max concept without the evidence to verify it. Ironically, the relative expression of the Taylor et al. $\dot{V}O_{2}$ plateau criterion defined as an increment $\dot{V}O_{2}$ that is < 50% of the stage increment is the one that should have been further tested by future research (see Part-2 of this series).

Mitchell et al. (1958) adopted a more stringent $\dot{V}O_{2}$ increment $\dot{V}O_{2}$ plateau criterion (<54 mL·min^−1^), yet as previously explained, there is concern over the accuracy of their $\dot{V}O_{2}$ measures. Such flaws make their reporting of how 72% of their 65 participants attained the $\dot{V}O_{2}$ plateau criterion challenging to interpret. Nevertheless, the lack of critical reflection by Mitchell et al. (1958) on the application and interpretation of the concept of a $\dot{V}O_{2}$ plateau at $\dot{V}O_{2}$ max further reinforced the narrative on this topic. What was needed at this time was the question and related theory for why all subjects were unable to attain a $\dot{V}O_{2}$ plateau!

It is a mystery that historical references of prior research pertinent to the $\dot{V}O_{2}$ max and $\dot{V}O_{2}$ plateau at $\dot{V}O_{2}$ max concepts continue to ignore the results of Wyndham et al. (1959) and Astrand and Saltin (1961a). Wyndham et al. (1959) were the first to reveal the non-linear complexities of the $\dot{V}O_{2}$ response (decreasing $\dot{V}O_{2}$ gain) to discontinuous incremental exercise, further revealing the data trend leading to what could be a $\dot{V}O_{2}$ plateau response. Similarly, Astrand and Saltin (1961a) extended this finding to document peak $\dot{V}O_{2}$ responses for multiple bouts of different exercise intensities, and in so doing revealed the presence of a $\dot{V}O_{2}$ plateau response in four of their five participants.

The other important concept to recognize about the origins of the $\dot{V}O_{2}$ plateau at $\dot{V}O_{2}$ max concept is that it was founded on discontinuous incremental exercise protocols. As such, the research that followed that of Hill and Lupton (1923) and Hill et al. (1924c) in this period of history was based on the in-built $\dot{V}O_{2}$ verification concept in addition to the priming effect of the prior moderate to more intense exercise bouts to multiple systems of physiology and muscle metabolism.

The consequence of this collection of research leading into the 1960s was that there was an underlying acceptance of the concept of a $\dot{V}O_{2}$ plateau at $\dot{V}O_{2}$ max, but this was not based on the research of Hill and Lupton (1923), Taylor et al. (1955) or Mitchell et al. (1958). Instead, as previously stated, the most important results for affirming the $\dot{V}O_{2}$ max and the $\dot{V}O_{2}$ plateau from this era came from Wyndham et al. (1959) and Astrand and Saltin (1961a). Yet such research also documented the wide individual variability in the changing $\dot{V}O_{2}$ gain of whole-body incremental exercise to contractile failure, though no discussion of what this meant to the concept of a $\dot{V}O_{2}$ plateau at $\dot{V}O_{2}$ max was evident in the literature of this era.

The added benefits of the collective of this period of research was that the $\dot{V}O_{2}$ max and $\dot{V}O_{2}$ plateau concepts were pertinent to both over-ground running, treadmill running, and cycle ergometry exercise, and that such discontinuous incremental exercise testing can occur on the same day with 10 min of recovery separating each exercise bout. Most importantly were the results of Astrand and Saltin (1961a) who, even though for a small number of participants (n = 5), introduced evidence on two crucial issues. First, there may be no singular relative non-steady state exercise intensity (or exercise time to failure) that can be applied as a discontinuous bout to all individuals to detect $\dot{V}O_{2}$ max. Second, the exercise intensity that induces the highest $\dot{V}O_{2}$ response across different discontinuous exercise bouts should cause volitional exhaustion in approximately 5 min, not 3 min.

Finally, the multiple theories developed and presented in this review direct scientists to the needed research that has largely been overlooked to current times. While further clarity on this issue is presented in Parts-2, -3 and -4 of this series, it remains reasonable to claim that further research is needed to compare $\dot{V}O_{2}$ max results between different discontinuous and continuous exercise protocols, for different exercise modes, for different recovery time periods and between different participant populations (e.g., trained, untrained, unhealthy). Critical thought and related commentary are also needed on whether the expectation of a $\dot{V}O_{2}$ plateau is needed for identifying $\dot{V}O_{2}$ max, or if in the absence of a $\dot{V}O_{2}$ plateau, the acceptance of secondary criteria to use as a proxy for the attainment of $\dot{V}O_{2}$ max is valid. Nevertheless, these issues transition into and are highly influenced by the content to be presented in Parts-2 and -3 of this review series, which span the time-periods for the transitions from discontinuous to continuous incremental exercise protocols, along with the improved temporal resolution of $\dot{V}O_{2}$ measurement using breath-by-breath technologies. The future Parts to this series will also reveal the results of more mechanistic research to improve understanding of the determinants to $\dot{V}O_{2}$ max and the presence, or not, of a $\dot{V}O_{2}$ plateau.

## Abbreviations

O_2_, gaseous oxygen; $\dot{V}O_{2}$ , the rate of O_2_ consumption; $\dot{V}O_{2}$ max, maximal rate of $\dot{V}O_{2}$ ; $\dot{V}O_{2}$ plateau, when close to exhaustion during incremental exercise, the inability to increase $\dot{V}O_{2}$ beyond a pre-established criterion value despite a further increase in exercise intensity; Δ, delta (change score); $\dot{V}O_{2}$ gain, the Δ $\dot{V}O_{2}$ /Δexercise intensity.
